# Uncovering the knowledge gap: A web-based survey of healthcare providers’ understanding and management of dengue fever in East Azerbaijan, Iran

**DOI:** 10.1371/journal.pone.0305528

**Published:** 2024-06-21

**Authors:** Madineh Abbasi, Morteza Zaim, Mahmood Moosazadeh, Mahasti Alizadeh, Abbasali Dorosti, Simin Khayatzadeh, Seyed Hassan Nikookar, Ahmad Raeisi, Fatemeh Nikpoor, Abdolreza Mirolyaie, Behrooz Naghili Hokmabad, Ahad Bazmani, Farzad Kaveh, Somayeh Azimi, Ahmadali Enayati

**Affiliations:** 1 Infectious and Tropical Diseases Research Center, Tabriz University of Medical Sciences, Tabriz, Iran; 2 Department of Medical Entomology & Vector Control, School of Public Health, Tehran University of Medical Sciences, Tehran, Iran; 3 Faculty of Health, Department of Medical Entomology and Vector Control, Health Sciences Research Center, Mazandaran University of Medical Sciences, Sari, Mazandaran, Iran; 4 Non-Communicable Disease Institute, Gastrointestinal Cancer Research Center, Mazandaran University of Medical Sciences, Sari, Iran; 5 Health Management and Safety Promotion Research Institute, Social Determinants of Health Research Center, Tabriz University of Medical Sciences, Tabriz, Iran; 6 Department of Anesthesiology, Tabriz University of Medical Sciences, Tabriz, Iran; 7 Province Health Center, Tabriz University of Medical Sciences, Tabriz, Iran; 8 Department of Vector Borne Diseases, Communicable Diseases Control, Ministry of Health, Tehran, Iran; 9 Institute for Environmental Research, Tehran University of Medical Sciences, Tehran, Iran; 10 Department of Health Education and Promotion, Tabriz University of Medical Sciences, Tabriz, Iran; 11 Department of Medical Entomology and Vector Control, School of Public Health and Health Sciences Research Center, Mazandaran University of Medical Sciences, Sari, Iran; Pasteur Institute of Iran, ISLAMIC REPUBLIC OF IRAN

## Abstract

**Background:**

Dengue fever (DF) is increasingly recognized as one of the world’s major mosquito-borne diseases and causes significant morbidity and mortality in tropical and subtropical countries. Appropriate and timely diagnosis and risk stratification for severe disease are crucial in the appropriate management of this illness. Healthcare providers (HCPs) play a key role in dengue fever diagnosis, management and prevention. The present study was conducted to determine the knowledge, attitudes and practices (KAP) among HCPs in East Azerbaijan Province, Iran.

**Methods:**

A cross-sectional survey among 948 HCPs, using a structured questionnaire, was conducted in East Azerbaijan Province from May to July 2022. Data analysis was undertaken using descriptive methods, the Chi-square test or Fisher’s exact test, and logistic regression. A P-value <0.05 was considered for statistical significance.

**Results:**

Out of the 948 (68.5% female) respondents, 227 were physicians and 721 were health professionals. The knowledge level of DF was found to be largely inadequate in the present study population (80.4%). The physician vs. health professional were a significant factor in differentiating attitude scores. The mean practice score regarding DF prevention and control measures among respondents was 8.40±1.97.

**Conclusion:**

The findings call for urgent continuous education and training courses to increase KAP levels and increased capacity and capability for DF prevention and control. This is of outmost importance for the first point of care of DF patients.

## Introduction

Dengue fever (DF) is a rapidly spreading, mosquito-transmitted infection that poses a serious public health threat in tropical and subtropical regions. In the past few decades, cases of DF have increased by over 30-fold, resulting in approximately 96 million symptomatic cases and 40,000 deaths annually [1–4]. World Health Assembly raised concern that dengue may become a public health emergency of international concern [5]. Dengue Fever virus is transmitted to humans by *Aedes aegypti* and *Aedes albopictus*. Although many infected individuals do not show any symptoms, disease severity ranges from mild acute fever with headache and muscle/joint pain to severe symptoms such as ecchymosis, pulmonary issues, cardiac and hepatic problems with a plasma leakage syndrome resulting in hypovolemic shock and haemorrhage [6–9]. Repeated infection increases the risk of life-threatening forms of the illness [10]. Without proper treatment, the mortality rate of DF can be as high as 20%, but this can be reduced to less than 5% with early diagnosis and treatment [11,12].

The distribution range of *Aedes aegypti* and *Aedes albopictus* is expanding throughout the world in recent decades. Even though Iran is not considered an endemic country of dengue fever as yet, due to the establishment *Aedes aegypti* and *Aedes albopictus* in the north and south of Iran, [13–15] and because of huge travel and trade between the north and south of the country, there is a potential for the expansion of the distribution range of these vectors throughout the country. Also, travel and trade with dengue-endemic countries (India, China, Thailand, Malaysia, Pakistan, Saudi Arabia, Afghanistan) pose Iran at continuous risk of invasion of vector species and epidemics of the diseases they transmit through virus circulation by imported cases [7,11,16–20]. The first case of DF in Iran was recorded in a patient who had previously visited Malaysia in 2008. Subsequently, 15 cases were confirmed to be positive through serological testing that eight cases linked to individuals who had travelled to Malaysia, India, and Thailand, while the remaining seven cases were not associated with foreign travel [7,21].

The widespread transmission of DF is believed to be influenced by environmental and socio-demographic factors such as unplanned urbanization, increased movement of people and goods, and climate and environmental changes [22]. According to one estimate, around 60 percent of the world’s population could be exposed to DF due to global climate change by 2080s [23]. The significant potential for explosive outbreaks of DF and its increasing prevalence worldwide cannot be ignored [24]. DF is endemic in many parts of the world, particularly Central and South America, South and South-eastern Asia, the Western Pacific region, Africa, and Australia [25–27]. Asia continues to have the highest dengue cases, and the high mortality and morbidity rates reported annually in Southeast and the tropical regions of Asia have made countries in this region the epicentre of dengue outbreaks [28]. Increased cases of DF have also more recently been reported from countries neighbouring Iran such as Afghanistan, Pakistan, Oman, Saudi Arabia and Yemen [29–31].

HCPs are in a unique position to influence the course of DF; not only from their responsibility regarding patient care, but also for community prevention [32]. Studies undertaken on the knowledge, attitude and practice show that healthcare professionals’ perception regarding DF can vary significantly: a study in Ethiopia found only 49% had moderate levels of knowledge [9]; while in Taiwan over 80% had deficits regarding mosquito-transmitted diseases including malaria, yellow fever and DF [33]. According to Ekra’s study, three quarters of the study subjects knew that DF was a sequential disease with one fifth showing good knowledge & diagnostic practice [34].

Although vaccination can have a big role in reducing the global burden of dengue, developing a safe and effective vaccine proved to be extremely difficult until recently when Dengvaxia, a tetravalent dengue vaccine (CYD-TDV), has been approved and licensed in 20 countries. It has the limitation that should only be administered to individuals with previous dengue infection and living in endemic areas. Therefore, other means of prevention and control of the disease are still the mainstay. As health care providers are in the frontline of prevention and control or otherwise management of dengue, health education to HCPs is important for adequate undertaking of these tasks. Workshops have been held for HCPs and it is important to assess the level of knowledge, attitude and practice among them to find out the adequacy of the education as well as revising the educational material should it deem necessary [35,36].

East Azerbaijan province shares common borders (through land, air, and rail) with a number of countries where invasive *Aedes* species exist. As such, there is a high chance to become infested by these vectors. In addition, there is a threat of the arrival of invasive mosquitoes from the southern regions of Iran. Therefore, this study was designed and undertaken to assess the KAP among health care providers in this province, the results of which may help policymakers to enhance the prevention, control, diagnosis and management of dengue and other *Aedes*-borne diseases.

## Methods

### Study design and population

**Fig 1:**
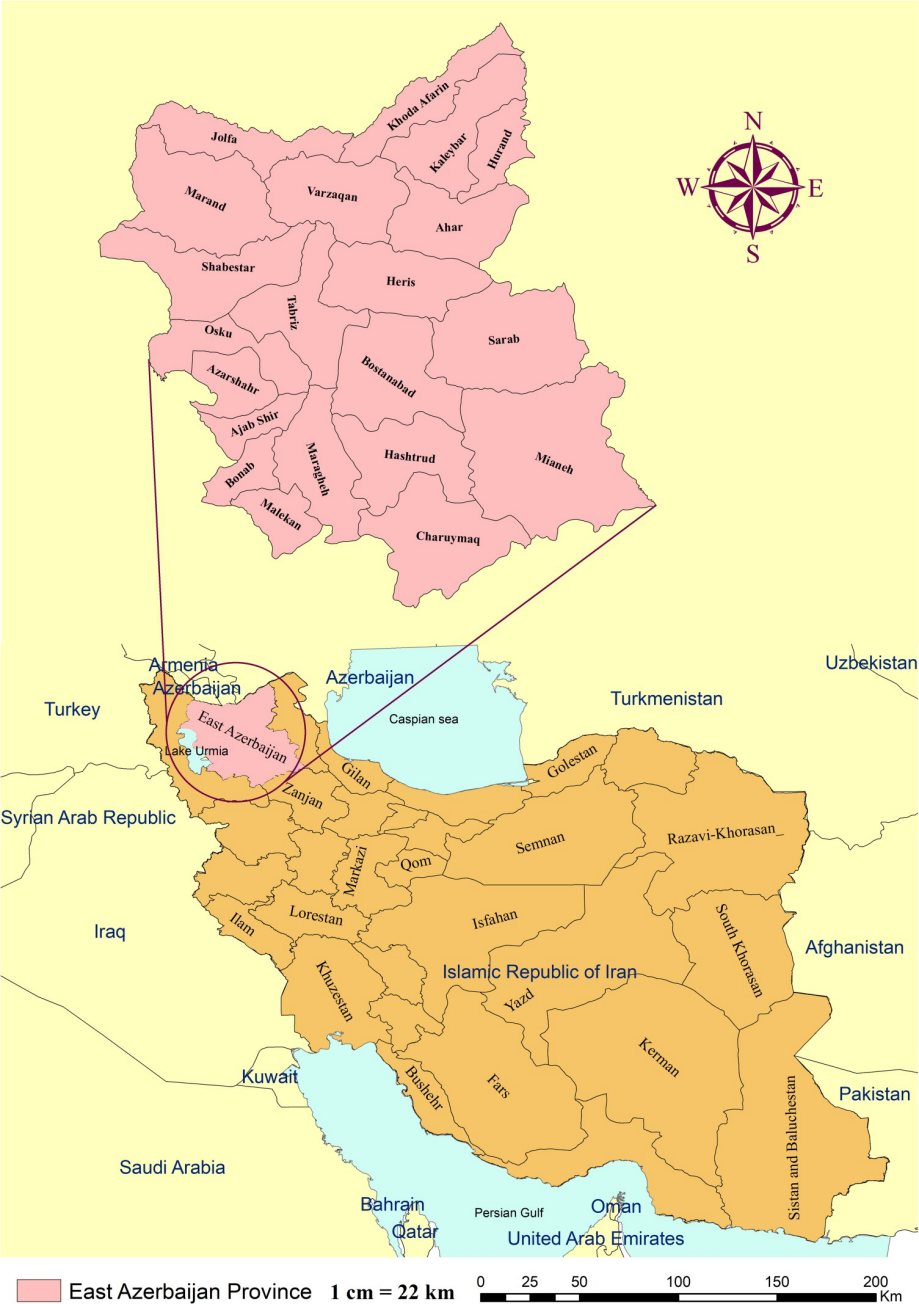
Map of study area, counties of East Azerbaijan northwest of Iran.

This descriptive cross-sectional survey was conducted from 1 May to 30 July 2022 among HCPs working in public and private and health centers of East Azerbaijan Province. The province lies in the northwest of Iran and shares its borders with Armenia, Turkey and the Republic of Azerbaijan (Fig 1).

The population under study was the HCPs who were involved in the prevention, control, diagnosis and management of dengue. They include four groups: a) Physicians comprised general practitioners as well as specialists in infectious diseases, internal medicine, emergency medicine, and paediatrics; b) Health professional staff includes experts in diseases control and management, environmental health, and health education; c) Central provincial level health professional staff, individuals within the Health and Treatment Deputies of the East Azerbaijan university and the health networks of counties; d). Experts related to the Ministry of Health and Medical Education involved in national decision-making and management. Peripheral level health professionals are individuals who provide services at comprehensive health centers to the general public and direct visitors. The level of education of these health professionals are BSc and MSc degrees. Our study population included general practitioner and specialist, health professional staff-central level and health professional staff-peripheral level.

The size of target population included 2500 general practitioner, 350 specialist, 490 Health professional staff-central level, and 2200 Health professional staff-peripheral level. The sample size was calculated based on literature on the same study subject and study groups [37]. Based on type 1 error = 0.05(95% confidence level), the type 2 error = 20% (power 80%), and using G, POWER software, the number of participants was estimated to be 625. Participants were recruited by using a convenience sampling method from health centers of East Azerbaijan Province. During the 3 months of the study, 948 men and women completed the informed consent and web-based test. This number of respondents was more than our required sample size, so the analysis of the results started with this number.

It should be noted that of the 948 people who participated in the study, there were 150 general practitioners, 77 specialists, 354 Health professional staff-central level, and 367 Health professional staff-peripheral level.

### Study instrument

The validity and reliability of the questionnaire was assessed in a pilot study before it was distributed to the participants. The Cronbach’s alpha coefficient for the developed questionnaire was 0.79, indicating acceptable internal consistency, the results of the study on the validity and reliability of the instrument was published already [38] and was used elsewhere [37].

The study instrument comprised of a questionnaire composed of four parts: demographic information, knowledge, attitude and practice. The knowledge domain contained 50 questions about signs and symptoms, transmission routes, clinical management of the disease, prevention and control. Each correct response was associated with one score and each incorrect response with zero score. Participants who scored 70% or higher (≥35 correct answer) were considered to have a high knowledge; those who scored lower than 70% (0–34 correct answer) were considered to have low knowledge. The attitude domain contained 14 questions about DF, answered on a 5-point Likert scale from strongly agree (5 point) to strongly disagree (1 point). Scores above 90% (score ≥63) were classified as "high". The practice domain consisted of 16 items regarding prevention and treatment that each correct response was associated with one score and each incorrect response with zero score. Scores above 70% (≥12 correct answer) were classified as "high" while those below 70% were deemed "low". These individual scores were combined according to the total number of questions in the questionnaire, resulting in a possible maximum score of 50 for knowledge, 70 for attitude, and 16 for practice (S1 File).

### Data collection

The questionnaire was created on Google Docs and shared through various communication channels such as phone messages, emails, WhatsApp, and social media platforms including Facebook, Instagram, and LinkedIn to all study population. Participants were required to give their informed consent and read a short introduction before answering a series of questions that were presented in a sequential manner on their device screens.

### Statistical analysis

Statistical analysis was performed using the Statistical Package for the Social Sciences (IBM SPSS Statistics for Windows, Version 24). Descriptive statistics were utilized to illustrate characteristics of participants, level of their knowledge as well as their attitude and practice. The Chi-square test was conducted to compare KAP scores with different variables, while Fisher’s exact test was used when more than 20% of cells had expected counts below 5. Stepwise (univariate and multivariate) regression was used to identify independent predictors of KAP score. Logistic regression analysis included all demographic variables as explanatory variables with regard to KAP domains as outcome variables. To determine confounding factors, the difference between adjusted odds ratio (aOR) in multivariate analyses and crude odds ratio (OR) in univariate analyses were compared for a particular predictor variable affecting KAP domain. A p-value of less than 0.05 indicated statistical significance.

### Ethical statement

The ethical approval of this study has been obtained (Ethics Code: IR.TBZMED.VCR.REC.1399.405). Prior to completing the questionnaire, informed online consent was obtained from all participants. Those who did not wish to take part were given the option to opt out. Throughout the research process, subjects’ responses were kept confidential.

## Results

### Study population characteristics

Of the 948 (68.5% female) respondents, 227 were physicians and 721 were health professionals. The mean ± SD age of participants was 39.34 ± 10.10 years. Internal medicine, infectious diseases and paediatrics were the leading specialties among physicians. The primary source of DF-related information reported by 41.8% of the respondents was academic training and workshops. Table 1 displays the demographic characteristics of study participants.

**Table 1:** Demographic characteristics of HCPs in KAP study of dengue fever in East Azerbaijan, Iran (n = 948).

Characteristics	Frequency	Percent
**Job**		
**Health professional staff-central level** **Physician ‐ general practitioner** **Physicians ‐ specialist** **Health professional staff-peripheral level** **Total**	354 150 77 367 948	37.3 15.8 8.1 38.7 100.0
**Gender**		
**Female** **Male** **Total**	649 299 948	68.5 31.5 100.0
**Age (years)**		
**<30** **30–39** **40–49** **≥50** **Total**	200 281 295 172 948	21.1 29.6 31.1 18.1 100.0
**Workplace**		
**Private** **Public** **Total**	168 780 948	17.7 82.3 100.0
**sources of information About dengue**		
**Academic training, workshops** **Social media** **Others** **Total**	396 332 220 948	41.8 35.0 23.2 100.0

### Knowledge of respondents on DF

The mean knowledge score was 30.90±4.35 and 80.4 percent of participants had a low level of knowledge about DF prevention and control. There was a significant difference between job groups in terms of their level of knowledge regarding symptoms (P = 0.028) and clinical management (P<0.001). It seems that most of the respondents were aware of the general symptoms of DF such as fever (95.4%), headache (91%), and rash (77.8%). Moreover, the majority of healthcare professionals were aware that the disease was transmitted by *Aedes* mosquitoes (93.6%). Despite this, only about 13% knew that these mosquitoes bite during the daytime. Regarding prescribing corticosteroids for treating the disease, 62.7% gave a correct answer with specialist physicians providing more accurate responses than general physicians, a difference that was statistically significant (P<0.001).

(Fig 2).

**Fig 2:**
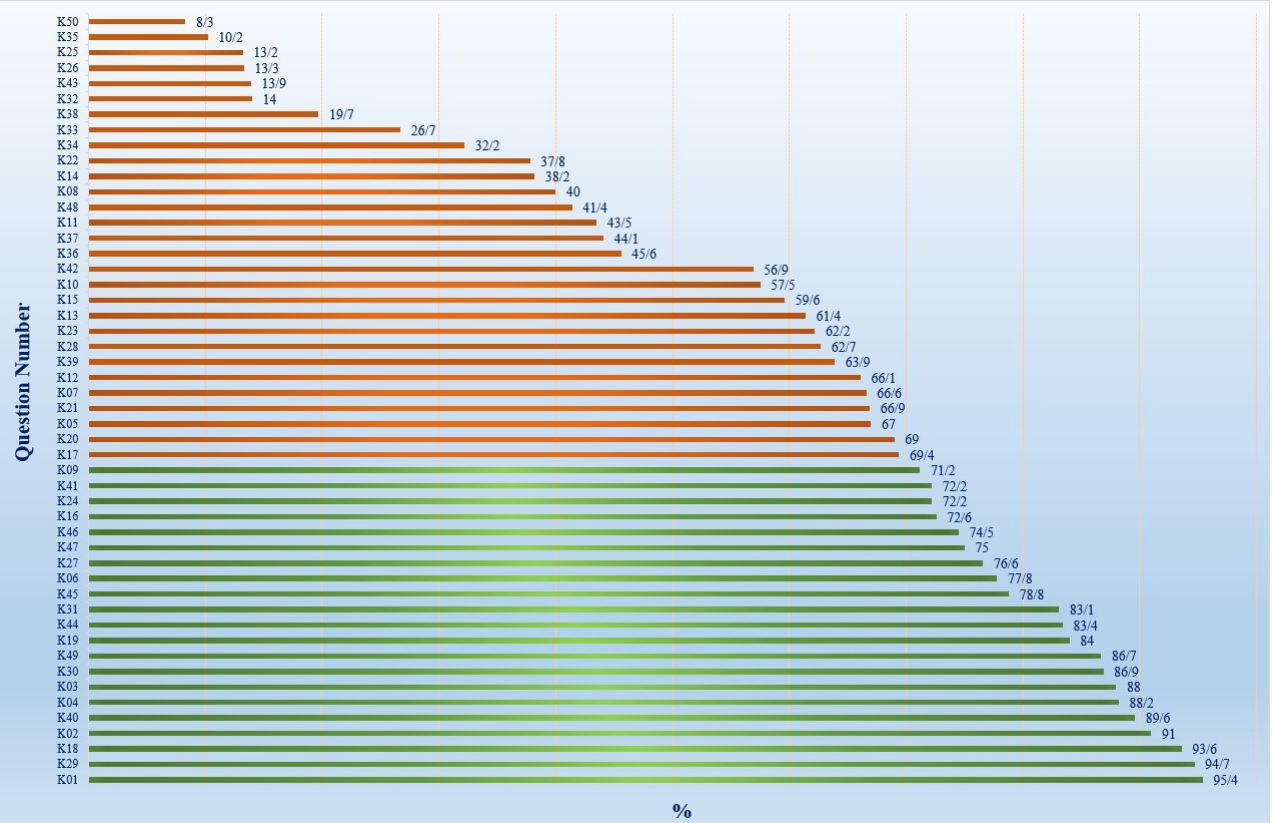
Knowledge level about dengue fever in East Azerbaijan province, Iran (n = 948). Green column = high knowledge, red column = low knowledge.

### Attitude toward DF

The average attitude score towards DF prevention and control was 54.23 ±6.49%. Most respondents agreed or strongly agreed that DF infection is dangerous (83.8%) and preventable (84.5%). Less than one-fifth (19.7%) of the participants strongly agreed on the appropriate diagnostic methods for DF virus infection, which are PCR and ELISA. Job was seen to be a significant factor in differentiating attitude scores; no other demographic variables demonstrated this relationship.

(Fig 3).

**Fig 3:**
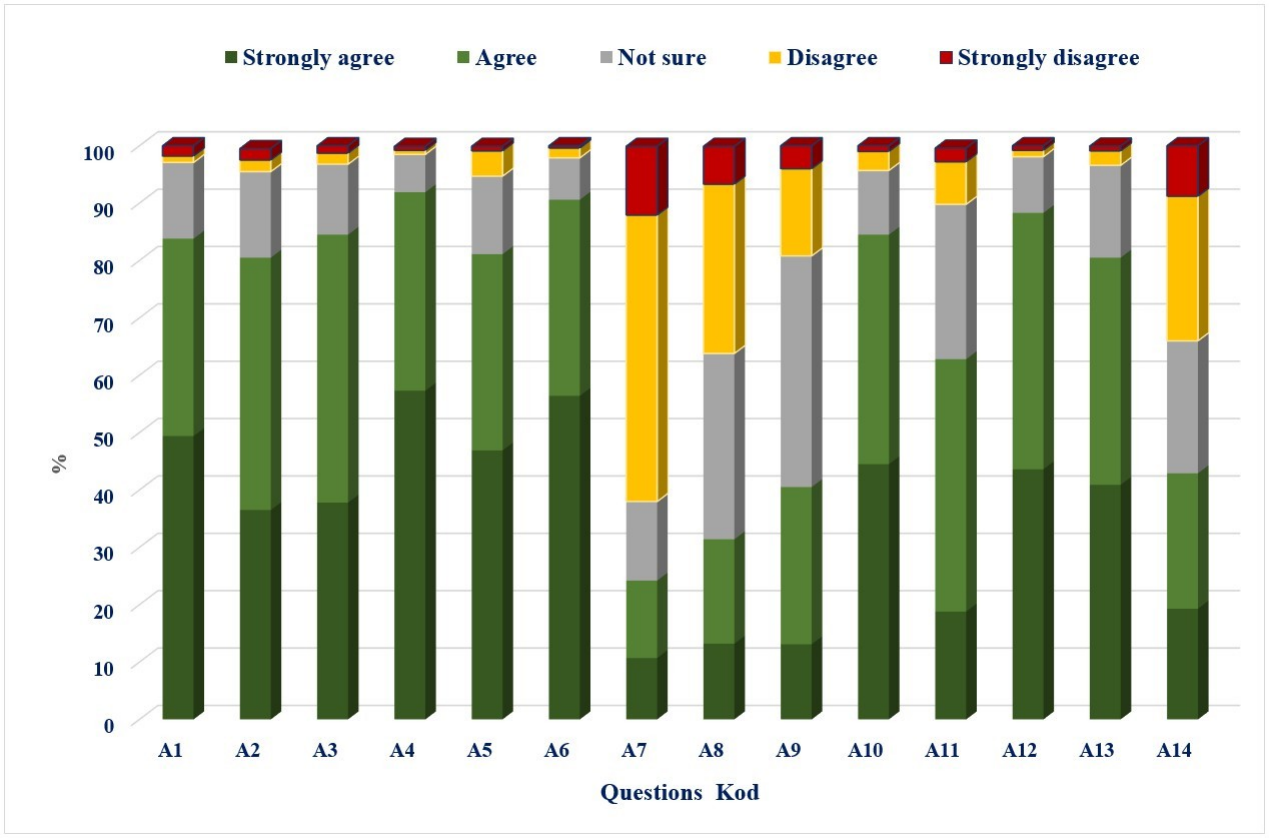
Attitude level about dengue fever in East Azerbaijan Province, Iran (n = 948).

### Prevention and control practices

The mean practice score among respondents was 8.40±1.97 in regard to prevention and control measures against DF. The lowest correct answers given involved immediate reporting of suspected disease cases (30.2%). Among Physician ‐ specialist, the lowest correct answers were observed; however, this difference was not statistically significant. Common good practices included use of mosquito coils and fans at home to reduce vector-human contact.

(Fig 4).

**Fig 4:**
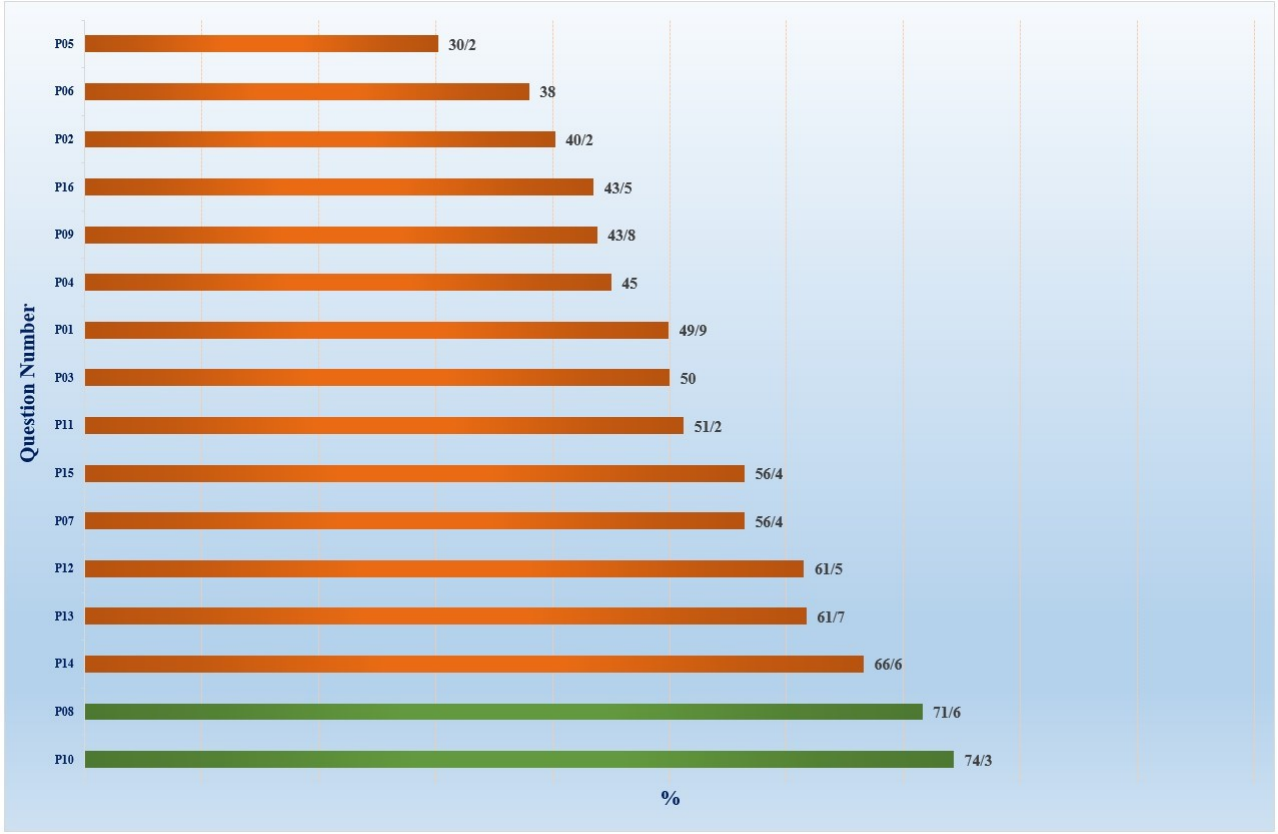
Practice level about dengue fever in East Azerbaijan Province, Iran (n = 948). Green column = High Practice, Red column = Low Practice.

### The effect of demographic factors on the KAP of DF

The correlation between KAP regarding DF and demographic factors was assessed with a binary logistic regression. Through univariate analysis of the associations between KAP and demographic variables, it was determined that males had increased odds of having higher KAP levels compared to females (Odds Ratio: 1.48, 95% CI: 1.06 to 2.07, P = 0.019). Participants from public sectors had higher odds of having high KAP scores than those from private ones. After excluding insignificant predictor factors (P>0.25), a multivariate model revealed that job type was an independent factor for predicting knowledge and attitude towards DF. Participants aged 30–39 years were 1.58 times more likely to have higher attitude scores compared to those aged under 30 years (OR: 1.58; 95% CI: .81 to 3.07); however, this difference was not statistically significant. Additionally, no association was observed between practice scores and socio-demographic variables (Table 2).

**Table 2:** Univariate and multiple logistic regression analysis showing the predictors of knowledge, attitude and practice levels (high/low).

Independent variable	Knowledge level				Attitude level				Practice level			
	Univariate		Multivariate		Univariate		Multivariate		Univariate		Multivariate	
	OR (95% CI) P‑value		AOR (95% CI) P‑value		OR (95% CI) P‑value		aOR (95% CI) P‑value		OR (95% CI) P‑value		aOR (95% CI) P‑value	
Job Health professional staff-central level(R) Health professional staff-peripheral level Physician ‐ General practitioner Physician ‐ specialist	1 .66 (.45-.97) 1.32 (.85–2.06) 1.06 (.58–1.93)	.037 .215 .830	1 .60 (.40-.90) 1.23 (.75–1.97) .94 (.49–1.78)	.014 .411 .857	1 2.11(1.23–3.61) 1.12 (.51–2.42) 1.81 (.77–4.25)	.006 .774 .168	1 2.19 (1.27–3.70) 1.12 (.50–2.50) 1.90 (.77–4.65)	.005 .779 .158	1 1.28 (.72–2.27) 1.06 (.49–2.30) .82 (.27–2.44)	.391 .866 .721	1 1.38 (.77–2.46) 1.00 (.44–2.24) .76 (.24–2.36)	.271 .997 .636
**Sex** Female(R) Male	1 1.48 (1.06–2.07)	.019	1 1.25 (.87–1.80)	.219	1 1.51(.95–2.41)	.078	1 1.58 (.96–2.63)	.072	1 1.11 (.65–1.90)	.679	1 1.03 (.58–1.84)	.905
**Age (years)** <30 (R) 30–39 40–49 ≥50	1 1.63 (.99–2.67) 1.73 (1.06–2.82) 1.51 (.87–2.61)	.051 .026 .138	1 1.65 (.98–2.77) 1.61 (.96–2.67) 1.00 (.54–1.84)	.056 .066 .998	1 1.58 (.81–3.07) 1.17 (.59–2.33) 1.17 (.54–2.54)	.171 .642 .678	1 1.46 (.74–2.88) 1.10 (.54–2.22) 1.09 (.46–2.53)	.274 .778 .842	1 .79 (.38–1.63) .75 (.36–1.54) 1.26 (.60–2.64)	.530 .442 .531	1 .81(.39–1.70) .77 (.37–1.60) 1.60 (.71–3.58)	.592 .488 .252
**Workplace** Private (R) Public	1 .679 (.45–1.00)	.054	1 .64 (.41-.99)	.048	1 .70 (.36–1.35)	.288	1 1.47(.74–2.93)	.270	1 1.57 (.73–3.37)	.240	1 1.72 (.77–3.81)	.181

OR = Odds Ratio, AOR = Adjusted Odds Ratio, CI = Confidence Intervals, R: Reference category.

## Discussion

HCPs serve as the first line in prevention, control, diagnosis, notifying and managing DF cases, so their knowledge, attitudes and practices (KAP) are instrumental in prevention and early detection to reduce the spread and severity of DF. Regarding gender, the majority of the participants were females. Most of respondents in our study belonged to the age group <40 years; this suggests a lower response rate from senior HCPs. Younger HCPs are more technologically knowledgeable and experienced with online surveys. Compared to other studies on general population, a higher level of awareness is observed among participants in our study owing to their roles as healthcare professionals. Our respondents’ most common source for information related to DF was academic training and workshops; similar results were found by Ekra & Oche’s [25,34].

According to the present study, only a small proportion of the HCPs (19.6%) achieved a high knowledge score. This is unexpected considering the background of the study subjects and the fact that working in a tertiary health and training institution exposes them to continuous medical education in the form of seminars and in-service training. However, similar to these findings, lesser knowledge scores were reported among health workers in Tanzania, Ecuador, and Taiwan [32,39,40]. Also, in the Thayer’s study, physicians had basic knowledge but lacked clinical diagnosis and management and needed training [41]. The results of the study of Turkey as a non-endemic country were also similar to Iran [42]. This may indicate the need for more targeted on the job training for HCPs.

In this study, the highest percentage of lack of knowledge was related to the dimensions of prevention (88.6%) and transmission (77.4%), which was consistent with Ho’s study [32]. In another study conducted in Mazandaran, Iran, HCPs had higher knowledge associated with the transmission, clinical management, and prevention and control of dengue as compared with the results of our study [37]. This difference may be due to the non-endemic nature of DF in Iran, probably no significant attention has been paid to the increased risk of dengue and other non-indigenous arboviruses. However, it might also be the case that HCPs in Mazandaran Province have participated in more educational workshops and continuing medical education about dengue fever. This difference may also reflect the fact that more continuing medical education would increase the knowledge of the HCPs. Therefore, considering the report of the presence of *Aedes aegypti* and *Aedes albopictus* in the country [13,14] with a high risk of spreading throughout the country causing outbreaks and epidemics of arboviruses including dengue, it seems necessary to improve the knowledge of the HCPs by whatever means possible including but not limited to holding educational workshops, distributing posters and pamphlets in health and treatment centers, social media and etc.

Similar to the findings of this research (13.3%), in Khan’s study (13.3%) a low percentage of the participants were aware of the daytime biting habit of the dengue vectors [43] while in other studies, this proportion was quite different [25,37,39]. This misapprehension may lead healthcare providers to give patients incorrect preventive counsel. The majority of the respondents (62.7%) correctly knew that corticosteroids should not be administered to patients suspected of having dengue, which is consistent with Tomashek’s study (57%) and in line with WHO guidelines and a 2006 Cochrane review [44–46]. In a study in Turkey, 48% of the respondents mentioned the contraindication of corticosteroids for dengue patients [42].

Interestingly, 23.4% of participants in our study thought they should prescribe aspirin regardless of its consequences, such as decreased platelet function and severe bleeding—which is much higher than in Thayer’s study [41]. In other words, 76.6% had good knowledge of clinical management. In the Nikookar’s study [37], the majority of the respondents (82.1%) were aware of the avoidance of aspirin during dengue compared to other studies in Jamaica (29.8%) [47] and Sri Lanka (42%) [48].

Our study found that the majority of respondents (91.4%) had low attitudes toward DF. Oche’s study, and [25] and Nikookar’s study [37] reported high attitudes (93.2% (and (81%), while Yusuf’s study showed a largely neutral attitude at 46.7% [9]. This low attitude shows that most of the respondents did not appreciate the risk of dengue. Furthermore, the job was significantly associated with attitude level Physicians had a better attitude towards dengue fever; this may be because physicians were trained regarding the disease and have had more opportunities to participate in workshops about this disease and interact with patients with DF symptoms. The majority of healthcare professionals perceived DF as a dangerous disease and were supportive of prevention and control efforts. We also found that 62.9% of the participants believed that DF could be treated, which is consistent with Khan’s results [43]. In addition, our data showed a low attitude toward laboratory diagnosis of DF. This finding is in line with previous research from Puerto Rico, which revealed that only 6% of physician respondents knew about the laboratory tests used for diagnosis [44], and Koonisetty’s study, which indicated that only 21.5% were able to identify all available tests for DF [42]. Also Nikookar et al., reported that there was a low attitude in response to whether ELISA and PCR methods were suitable for dengue confirmation [37]. This appears to be because these participants had not been exposed to individuals affected by DF. Another reason may well be that they are not generally familiar with molecular and serological tests. It is necessary to have sufficient knowledge about the appropriate diagnostic tests, as early detection can reduce morbidity and mortality associated with DF.

In this study, most respondents (93.1%) did not have adequate practice for preventing DF. Data from other studies indicate a substantial discrepancy from these results [9,25]. 45% of the respondents stated that a survey of larvae, installation of ovitraps at the points of entry, entomological surveillance across the country, and disease surveillance are the most important strategies to prevent dengue when vectors of the disease are absent in the country. In the Nikookar’s study, this rate was 73% [37]. One possible explanation for this discrepancy could be that DF has not been prevalent in the study area, leading to a lack of awareness about prevention methods until the outbreak of the epidemic. Another possible explanation could be that there is no report of dengue fever vector in the study area. The results of this and other studies may serve the health authorities for evidence-based decision making in order to design and implement educational interventions aimed at promoting knowledge, attitude, and practice of the staff of comprehensive health services centers regarding dengue.

Although this study provides important baseline information about KAP, there are several considerations that should be taken into account when interpreting the results of the study. Convenience sampling was used, meaning that certain strata of participants may not have been included; additionally, the questionnaire only included general KAP characteristics, making it difficult to measure specific community correctness scores. Also, socio-economic questions were not included in the questionnaire, mainly because the respondents were more or less at the same level regarding these factors. Furthermore, social desirability bias cannot be completely ruled out as participants may have provided socially acceptable responses instead of their actual attitudes or practices. Also, while no DFV patients have been diagnosed in this province (like most provinces in the country), the evaluation of participants’ practice through this instrument should be interpreted with caution. Having said that, it should also be mentioned that the practice section is not restricted only to case diagnosis and management but as the whole of the country is at least in scenario one [49], preemptive source reduction and related practices are cruicial. Despite these limitations, this study provides important baseline information on the overall KAP of HCPs in East Azerbaijan regarding DF, guiding strategies and policies required to increase capacity and capability of health care providers in prevention and control of DF.

## Conclusion

In the present study, HCPs demonstrated inadequate knowledge, attitudes and poor practice when it comes to managing and preventing DF. Prevention and control as well as early diagnosis and timely and appropriate treatment, are critical public health measures needed to mitigate local transmission of DF and optimize clinical outcomes. Furthermore, due to increasing globalization caused by international trade and travel, there is an urgent need for comprehensive preparedness especially expanded training for HCPs in order to address common emerging tropical diseases.

Iran is particularly prone to DF due to its rather long common border with Pakistan (an endemic area) and other endemic countries through trade and travel. As such, there needs to be programs and activities implemented in order to develop the capacity of HCPs; this would enable them to identify possible missed cases of DF more easily and execute necessary surveillance or control strategies in the event of an outbreak. To this end, it is imperative that baseline knowledge of DF is established so that relevant interventions can be tailored specifically for areas where DF vectors might expand beyond its current boundary resulting to epidemics. This endeavor can only be accomplished with correspondingly trained managers, health planners, health care policymakers, and local government officials.

Therefore, more research should be conducted to better understand why there are discrepancies between studies and why certain HCPs disregard DF management protocols. This suggests that there is an urgent need for HCPs to receive proper training on all facets pertaining to DF in order to ensure that appropriate treatments are administered and that preventative measures are implemented effectively where necessary.

## Supporting information

S1 File The knowledge, attitudes, and practices (KAP) Questions about dengue fever in East Azerbaijan province, Iran. (DOCX)

S1 Graphical abstract Knowledge, attitude, and practice regarding dengue fever among healthcare providers: A web-based cross-sectional survey in East Azerbaijan, Iran. (DOCX)
