# Supplementary material for: Uncovering the knowledge gap: A web-based survey of healthcare providers’ understanding and management of dengue fever in East Azerbaijan, Iran
Source: PLoS One. 2024 Jun 21;19(6):e0305528. doi: 10.1371/journal.pone.0305528 (PMC11192336; doi:10.1371/journal.pone.0305528)
Supplement: S1 File — (DOCX) [file pone.0305528.s001.docx]

**Knowledge Questions about dengue fever in East Azerbaijan province, Iran (n=948)**

| **Question Number** | **Knowledge about dengue symptoms** |
| --- | --- |
| **K1** | **Is fever one of the symptoms of dengue?** |
| **K2** | **Is headache one of the symptoms of dengue?** |
| **K3** | **Is joint pain one of the symptoms of dengue?** |
| **K4** | **Is muscle pain one of the symptoms of dengue?** |
| **K5** | **Is eye pain one of the symptoms of dengue?** |
| **K6** | **Are skin rashes one of the symptoms of dengue?** |
| **K7** | **Is stomach pain one of the symptoms of dengue?** |
| **K8** | **Is diarrhea one of the symptoms of dengue?** |
| **K9** | **Is cough one of the symptoms of dengue?** |
| **K10** | **Is chest pain one of the symptoms of dengue?** |
| **K11** | **Is dizziness one of the symptoms of dengue?** |
| **K12** | **Is microcephaly considered a symptom of dengue?** |
| **K13** | **Are swelling of hands and feet and joint inflammations prominent symptoms of dengue?** |
| **K14** | **Is conjunctivitis a prominent symptom of dengue?** |
| **Knowledge about dengue transmission** | |
| **K15** | **Are flies vectors of dengue?** |
| **K16** | **Are ticks vectors of dengue?** |
| **K17** | **Do malaria vectors (*Anopheles*) transmit dengue?** |
| **K18** | **Are *Aedes* mosquitos vectors of dengue?** |
| **K19** | **Does dengue transmit by sex?** |
| **K20** | **Does person-to-person contact transmit dengue?** |
| **K21** | **Does blood transfusion transmit dengue?** |
| **Is dengue a(n) ... disease?** | |
| **K22** | **Urban** |
| **K23** | **Rural** |
| **When does dengue transmission by vectors mostly occur?** | |
| **K24** | **At night** |
| **K25** | **During the daytime** |
| **K26** | **Both day and night** |
| **knowledge of dengue Clinical management** | |
| **K27** | **Do you prescribe aspirin for dengue?** |
| **K28** | **Do you prescribe corticosteroids for dengue?** |
| **K29** | **Is dengue a reportable disease?** |
| **Knowledge about prevention and control of dengue vectors** | |
| **K30** | **Is there a vaccine available for preventing dengue?** |
| **Where do dengue vectors breed and reproduce?** | |
| **K31** | **Human-made containers (buckets, used tires, drinking water barrels)** |
| **K32** | **Riverbanks** |
| **K33** | **Wide drainage system** |
| **K34** | **Rice fields** |
| **K35** | **Stagnant dirty water** |
| **K36** | **Animal feces** |
| **K37** | **Pastures and stables** |
| **Which of the following is used for personal prevention?** | |
| **K38** | **Insect repellents** |
| **K39** | **Long-sleeved white shirts and pants** |
| **K40** | **Window screens** |
| **K41** | **Personal hygiene measures** |
| **K42** | **Daily vitamin C intake** |
| **K43** | **Sleeping under bed nets at night** |
| **Which of the following is a method of vector control?** | |
| **K44** | **Eliminating or emptying water-holding containers** |
| **K45** | **Covering household water-storage containers** |
| **K46** | **Using chemical agents (larvicides) in water-holding containers** |
| **K47** | **Managing solid waste** |
| **K48** | **Regularly cutting grass** |
| **K49** | **Storing used tires under a roof or keeping them protected from rain with appropriate covers.** |
| **K50** | **Water management in rice fields** |

**Attitude level about dengue fever in East Azerbaijan province, Iran (n=948)**

| **Question Number** | **Attitude level about dengue fever** |
| --- | --- |
| **A1** | **Dengue is a dangerous disease.** |
| **A2** | **Iran is at risk of dengue vector invasion.** |
| **A3** | **Dengue is preventable.** |
| **A4** | **Control of dengue breeding places is a strategy to prevent this disease** |
| **A5** | **Tires, containers, and pots around homes are suitable breeding sites for dengue vectors.** |
| **A6** | **People must actively participate in controlling dengue vectors.** |
| **A7** | **Only the government is responsible for controlling dengue vectors.** |
| **The following methods are used to confirm dengue:** | |
| **A8** | **PCR** |
| **A9** | **ELISA** |
| **A10** | **Reporting cases of dengue is a national priority.** |
| **A11** | **Dengue is treatable.** |
| **A12** | **Follow-up visits for suspected dengue patients are necessary.** |
| **A13** | **It is necessary to perform complete blood counts at least every 48 hours in suspected dengue patients.** |
| **A14** | **Suspected dengue patients with warning signs and no access to complete blood counts should not start fluid therapy.** |

**Practice Questions about dengue fever in East Azerbaijan province, Iran (n=948)**

| **Question Number** | **Practice about dengue:** |
| --- | --- |
|  | **In non-establishment conditions of dengue vectors in the country, which of the following operations should be performed** |
| **P1** | **Larval surveys at entry points** |
| **P2** | **Installation of ovitraps at entry points** |
| **P3** | **Entomological Surveillance throughout the country** |
| **P4** | **Healthcare services** |
| **P5** | **Immediate reporting of suspected cases of the disease** |
|  | **In conditions of local transmission of dengue, which of the following operations are used:** |
| **P6** | **Insecticide spraying** |
| **P7** | **Larval control with insecticides** |
| **P8** | **Use of mosquito coils to reduce vector populations** |
| **P9** | **Installation of screens on windows to reduce vector populations** |
| **P10** | **Use of fans to reduce vector populations** |
| **P11** | **Sleeping under mosquito nets at night** |
| **P12** | **Encouraging public participation to reduce vector breeding sites** |
| **P13** | **Elimination of small water containers around homes to reduce vector populations** |
| **P14** | **Trimming grass around homes to reduce vector populations** |
| **P15** | **Use of insect repellents** |
| **P16** | **Covering household water-storage containers** |
