# Supplementary material for: Uncovering the knowledge gap: A web-based survey of healthcare providers’ understanding and management of dengue fever in East Azerbaijan, Iran
Source: PLoS One. 2024 Jun 21;19(6):e0305528. doi: 10.1371/journal.pone.0305528 (PMC11192336; doi:10.1371/journal.pone.0305528)
Supplement: S1 Graphical abstract — (DOCX) [file pone.0305528.s002.docx]

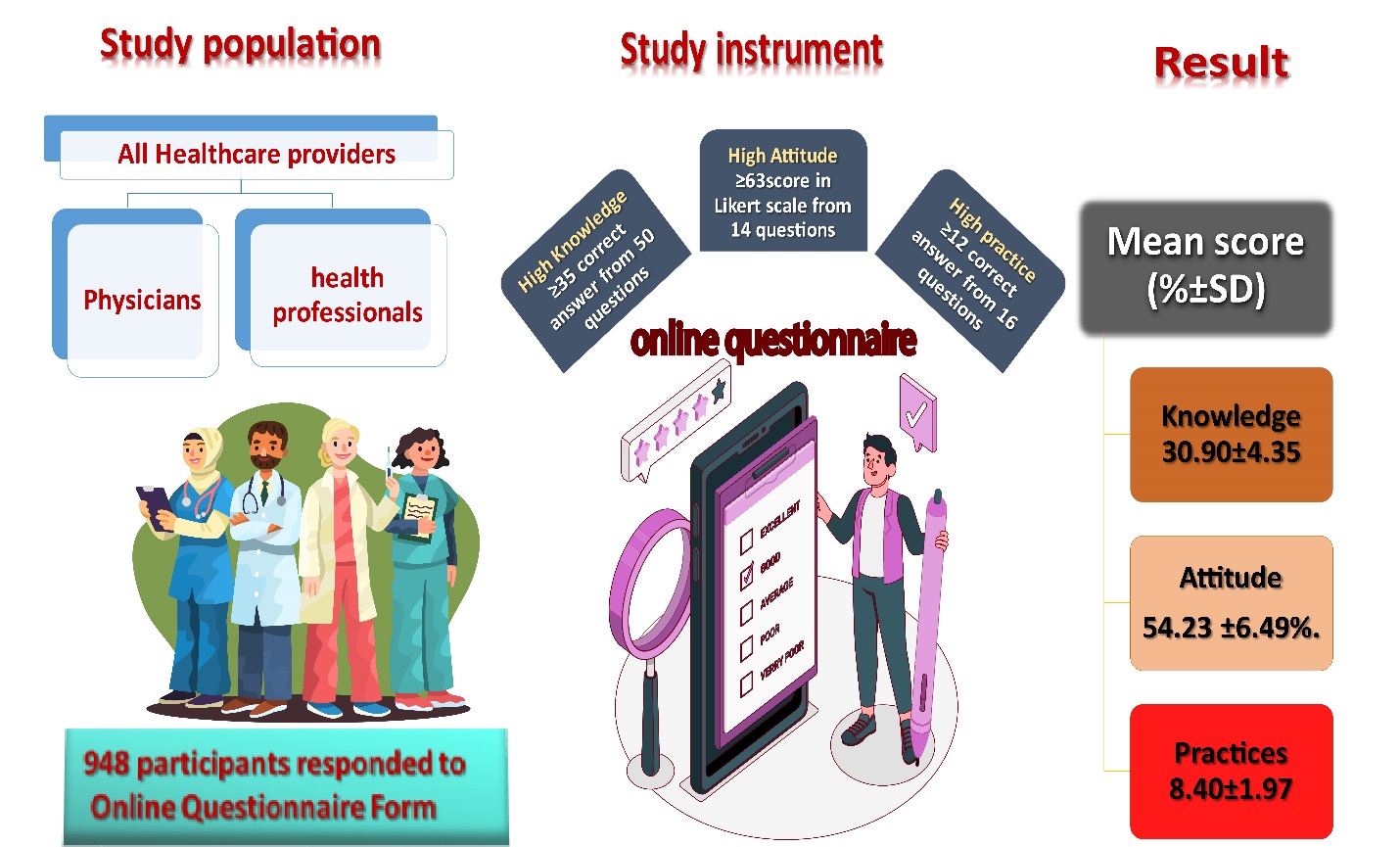


Graphical abstract: Knowledge, Attitude, and Practice Regarding Dengue Fever among Healthcare providers: A Web-Based Cross-Sectional Survey in East Azerbaijan, Iran
